# Rapid identification of pathogens associated with ventilator-associated pneumonia by Nanopore sequencing

**DOI:** 10.1186/s12931-021-01909-3

**Published:** 2021-12-10

**Authors:** Nan Wu, Piyush Ranjan, Changyu Tao, Chao Liu, Ence Yang, Bei He, John R. Erb-Downward, Shining Bo, Jiajia Zheng, Chenxia Guo, Beibei Liu, Lina Sun, Wei Yan, Meng Wang, Wenting Wang, Jianing Wen, Ping Yang, Lin Yang, Qiaoshan Tian, Robert P. Dickson, Ning Shen

**Affiliations:** 1 Department of Pulmonary and Critical Care Medicine, Peking University Third Hospital, Beijing, 100191 People’s Republic of China; 2 Department of Internal Medicine, University of Michigan Medical School, Ann Arbor, MI 48109 USA; 3 Department of Human Anatomy and Histology and Embryology, Peking University, Beijing, 100191 People’s Republic of China; 4 Department of Infectious Diseases, Peking University Third Hospital, Beijing, 100191 People’s Republic of China; 5 Department of Medical Bioinformatics, School of Basic Medical Sciences, Peking University Health Science Center, Beijing, 100191 People’s Republic of China; 6 Intensive Care Unit, Peking University Third Hospital, Beijing, 100191 People’s Republic of China; 7 Department of Laboratory Medicine, Peking University Third Hospital, Beijing, 100191 People’s Republic of China

**Keywords:** Ventilator-associated pneumonia, Endotracheal aspirate, Pathogen identification, Nanopore sequencing, Clinical identification method

## Abstract

**Background:**

Aetiology detection is crucial in the diagnosis and treatment of ventilator-associated pneumonia (VAP). However, the detection method needs improvement. In this study, we used Nanopore sequencing to build a quick detection protocol and compared the efficiency of different methods for detecting 7 VAP pathogens.

**Methods:**

The endotracheal aspirate (ETA) of 83 patients with suspected VAP from Peking University Third Hospital (PUTH) was collected, saponins were used to deplete host genomes, and PCR- or non-PCR-amplified library construction methods were used and compared. Sequence was performed with MinION equipment and local data analysis methods were used for sequencing and data analysis.

**Results:**

Saponin depletion effectively removed 11 of 12 human genomes, while most pathogenic bacterial genome results showed no significant difference except for *S. pneumoniae*. Moreover, the average sequence time decreased from 19.6 h to 3.62 h. The non-PCR amplification method and PCR amplification method for library build has a similar average sensitivity (85.8% vs. 86.35%), but the non-PCR amplification method has a better average specificity (100% VS 91.15%), and required less time. The whole method takes 5–6 h from ETA extraction to pathogen classification. After analysing the 7 pathogens enrolled in our study, the average sensitivity of metagenomic sequencing was approximately 2.4 times higher than that of clinical culture (89.15% vs. 37.77%), and the average specificity was 98.8%.

**Conclusions:**

Using saponins to remove the human genome and a non-PCR amplification method to build libraries can be used for the identification of pathogens in the ETA of VAP patients within 6 h by MinION, which provides a new approach for the rapid identification of pathogens in clinical departments.

**Supplementary Information:**

The online version contains supplementary material available at 10.1186/s12931-021-01909-3.

## Background

Ventilator-associated pneumonia (VAP) refers to pneumonia that occurs after patients have been on mechanical ventilation (MV) for at least 48 h and up to 48 h after extubation [1]. VAP is a common and serious complication of MV patients, leading to increased mortality [1]. Studies have shown that timely and effective antibiotic treatment, which depends on the rapid identification of pathogens, can significantly improve the cure rate of patients with VAP and reduce the risk of disease deterioration and death [2–4]. Timely pathogenic detection plays a crucial role in the process of disease diagnosis and treatment [2–4]. In China, the most common pathogenic bacteria of VAP include *Pseudomonas aeruginosa* (*P. aeruginosa*), *Acinetobacter baumannii* (*A. baumannii*), *Klebsiella pneumoniae* (*K. pneumoniae*) and *Staphylococcus aureus* (*S. aureus*) [5–7].

At present, the most commonly used pathogen detection method in clinical practice is still bacterial culture, as in the middle of the twentieth century [7, 8]. However, bacterial culture requires 24–48 h, and it is not conducive to rapid and accurate identification of pathogens. In addition, according to our clinical experience, microbiology culture samples collected before the use of antibiotics are only obtained from a few patients, which will suppress the positive rate of culture results. Genomic identification of endotracheal aspirate (ETA), which is independent of culture, has become a new method for the rapid identification of pathogens. qRT-PCR and PCR-based FilmArray (R) panel methods can quickly identify pathogens, but these methods can only be used for specific pathogens and are not useful for the detection of unknown pathogens [9–13]. Second-generation sequencing technology has the advantages of high throughput and sequencing analysis for unknown pathogens, but it also has high requirements for experimental equipment and high costs, so sequencing is difficult to carry out in clinical laboratories [14]. Therefore, it usually takes 24 h or more for second-generation sequencing from sample extraction to result acquisition.

Nanopore sequencing, recognized as a third-generation sequencing method, can quickly identify DNA or RNA sequences in real time. MinION based on Nanopore sequencing technology can be used for DNA sequence detection with only the requirement of being connected to a laptop, and the detection results can be read and analyzed in real time, providing clinical departments with the ability to carry out pathogen genome detection [15, 16]. Although it has been used in several laboratories to test samples of the lower respiratory tract, its methodology is not unified, and the influence of different processing methods is also not clear [13, 17, 18].

Therefore, this study compared the detection efficiency of different methods for different pathogens and constructed a data analysis method suitable for clinical departments in Chinese hospitals based on local servers; this study thus provides guidance and suggestions for the selection of pathogen identification methods for VAP patients.

## Methods

### Patients and group definition

**Fig. 1:**
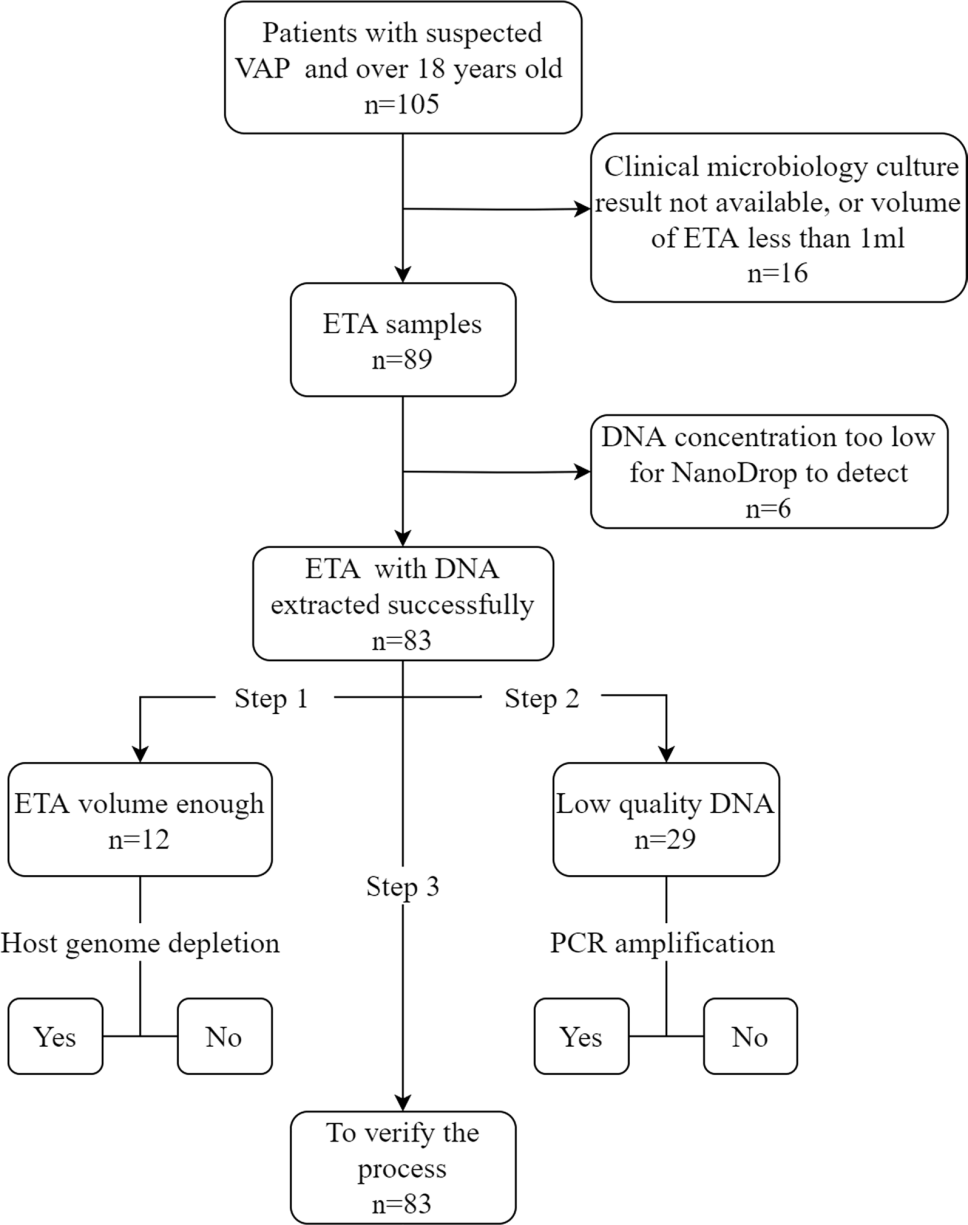
Sample Collection. A total of 105 patients with suspected VAP, and 16 of them had insufficient clinical information or samples. Enough information and samples were successfully acquired from 89 patients, but in 6 of the samples, an insufficient amount of DNA was extracted for further experiments. Step 1: 12 samples with enough volume were separated into two parts respectively, and DNA was extracted with or without host DNA depletion; Step 2: 29 low-quality DNA samples were used to build the library with and without PCR amplification. Step 3: all 83 DNA samples were extracted after host genome depletion and sequenced with the non-PCR amplification method for further analysis. VAP: ventilator-associated pneumonia; ETA: endotracheal aspirate

A total of 105 patients over 18 years old admitted to the respiratory intensive care unit, critical care unit and emergency department of Peking University Third Hospital (PUTH) from September 2019 to December 2020 who were experiencing MV for more than 48 h and were suspected of having VAP were collected. The criteria of VAP were defined according to the *Guidelines for the diagnosis and treatment of hospital-acquired pneumonia and ventilator-associated pneumonia in Chinese adults (2018 Edition)*: Chest X-ray or CT showing new or progressive infiltrating, consolidation, or ground glass shadows, accompanied by 2 or more of the following—temperature over 38 °C, purulent airway secretion, and white blood count above 4–10 × 10^9^/L [7]. Those suspected of having VAP were defined as meeting at least one of the 4 criteria. 16 of 105 recruited patients did not have complete clinical data or had insufficient samples; 89 samples were used to extract DNA, while 6 failed to provide enough DNA for further experiments. 83 patients had complete clinical data and sufficient samples and DNA and were finally admitted to this study (Fig. 1).

**Fig. 2:**
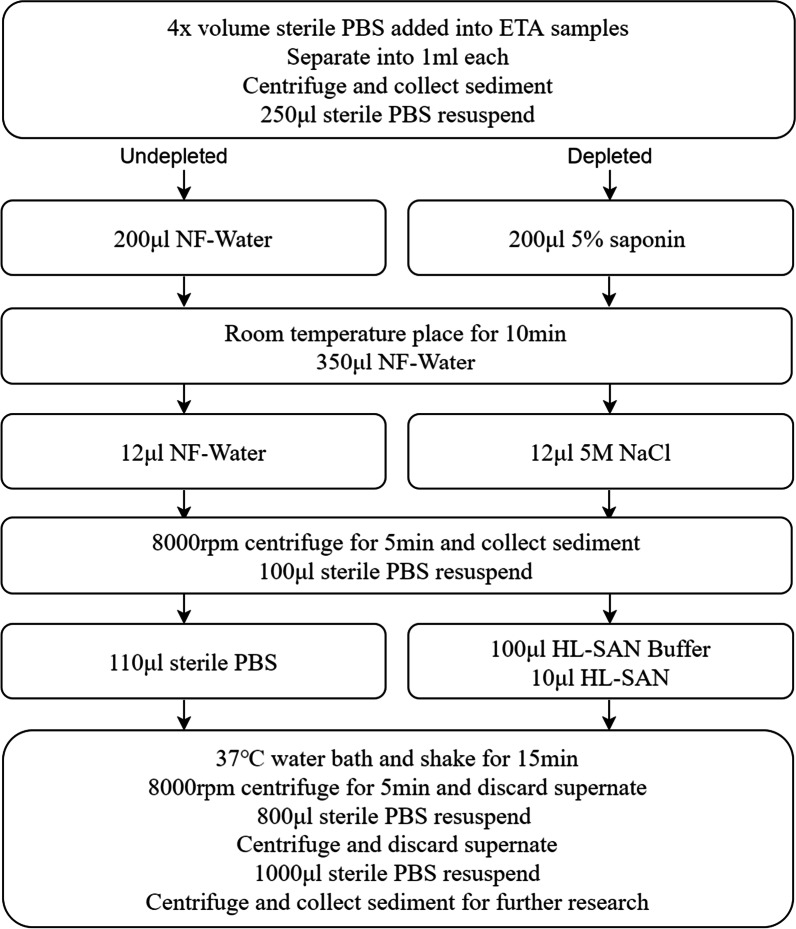
ETA host genome depletion protocol. Host genome depletion and non-depletion group was performed follow this protocol. ETA: endotracheal aspirate; PBS: phosphate buffered saline; NF-water: nuclease-free water; HL-SAN: heat-labile salt active nuclease

**Fig. 3:**
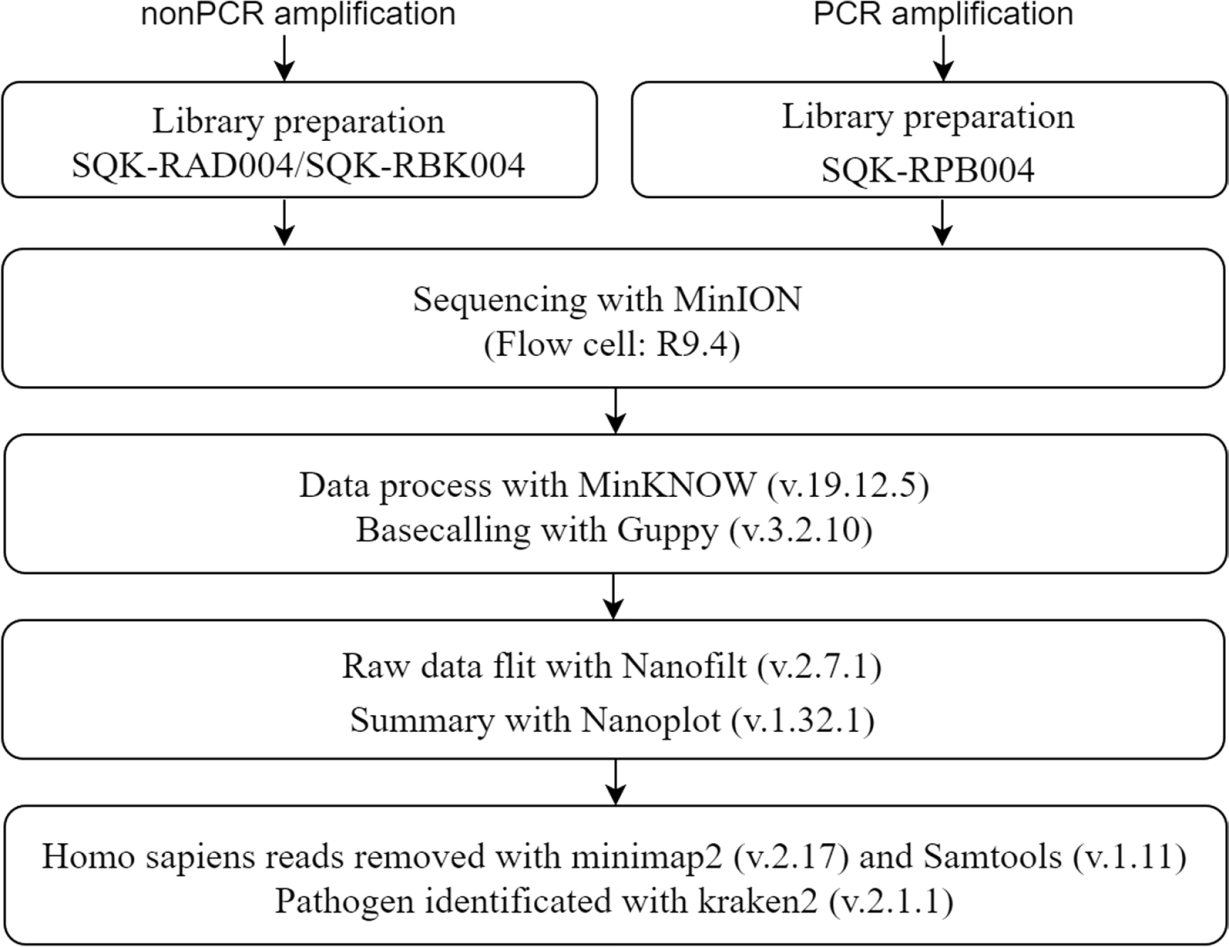
Metagenomic sequencing and analysis pipeline

To optimize the real-time pathogen detection process, we designed our study into 3 parts: Firstly, detect the efficiency of host genome depletion, 12 samples with enough volume were separated into 2 parts respectively, and DNA was extracted with or without host DNA depletion (Fig. 2); secondly, compare the differences in PCR amplification before library construction, 29 low-quality DNA samples (OD value 260/280 or 260/230 were out of 1.8–2.0) were used to build the library with and without PCR amplification (Fig. 3). In the end, all 83 DNA samples were extracted after host genome depletion and sequenced with the non-PCR amplification method (Fig. 1).

This study was approved by the Ethics Committee of Peking University Health Sciences (IRB00001052) and the Ethics Committee of Peking University Third Hospital (M20200352). All patients or families of unconscious patients were informed and agreed to participate in this experiment. The genetic resource management was proved by China Human Genetic Resources Management Office ([2021] GH3154).

### Sample collection

2 ETAs from patients suspected of having VAP were collected within 24 h: one was used for microbiology culture in the clinical laboratory of PUTH, and the other was taken to our laboratory for further research. A 4X volume of sterile PBS was added to the ETA sample, pipetted and aliquoted as 1 ml/tube. After centrifugation for 15 min at 8000 rpm, the sediment was collected, snap frozen in liquid nitrogen, and then stored at −80 °C (Fig. 2).

### Positive control strains collection

Standard strains of *S. aureus*, *A. baumannii*, *Stenotrophomonas maltophilia* (*S. maltophilia*), *P. aeruginosa*, *Streptococcus pneumoniae* (*S. pneumoniae*)*, Escherichia coli (E. coli)* *a*nd *K. pneumoniae* were obtained from American Type Culture Collection (ATCC) and the Clinical Culture Department of PUTH (Table 2). Monoclonal colonies were selected after overnight culture, dissolved in bouillon broth, shaken at 37 °C for 8 h, and centrifuged at 13,000 rpm at 4 °C for 1 min. The supernatant was disposed, and the pellet was resuspended in normal saline to make a suspension of 4.5 McFarland (McF). Then, samples were divided into 1 ml/tube and centrifuged at 13,000 rpm at 4 °C for 1 min. The sediment was collected, snap frozen by liquid nitrogen and stored at −80 °C.

### Negative control collection

Sterile saline solution was collected by aspiration through the sputum aspirator as a negative control, and the negative control was processed in parallel with the study samples.

### Host depletion with saponin

Sediments were resuspended in 250 μl of sterile PBS, and 200 μl of 5% saponin (S0019, Tokyo Chemical Industry, Tokyo, Japan) was added, followed by pipetting. Samples were placed at room temperature for 10 min before 350 μl of nuclease-free water (NF-water) was added and incubated for another 30 s. Then, 12 μl of 5 M NaCl was added and the tubes inverted. Next, the samples were centrifuged at 8000 rpm at 4 ℃ for 5 min, the supernatant was discarded, and the sediment was resuspended in 100 μl of sterile PBS. 100 μl of heat-labile salt active nuclease (HL-SAN) Buffer (100 mM MgCl2 in 5 M NaCl) and 10 μl HL-SAN DNase (25,000 units, 70910-202, Articzymes, Tromso, Norway), were added, and the samples were shaken at 37 °C for 15 min. Finally, the samples were centrifuged at 8000 rpm at 4 °C for 5 min, the supernatant was discarded, and the sediment was washed with 1000 μl of sterile PBS two times. The same procedure was used in the undepleted group, but all reagents were replaced by NF-water (Fig. 2).

### DNA extraction

BSCC45S1E kits and GenePure Pro (Bioer Technology, Hangzhou, Zhejiang, China) were used for DNA extraction. Lysozyme was dissolved in TET buffer and mixed by shaking. A 180 μl mixture was added to each sample and incubated for 30 min at 37 °C after shaking. Then, 20 μl of Proteinase K and sample were added to columns 1 and 7 of the kit and placed into the machine. DNA concentration and purity were determined by a NanoDrop after extraction.

### Library construction, sequencing and data analysis

**Fig. 4:**
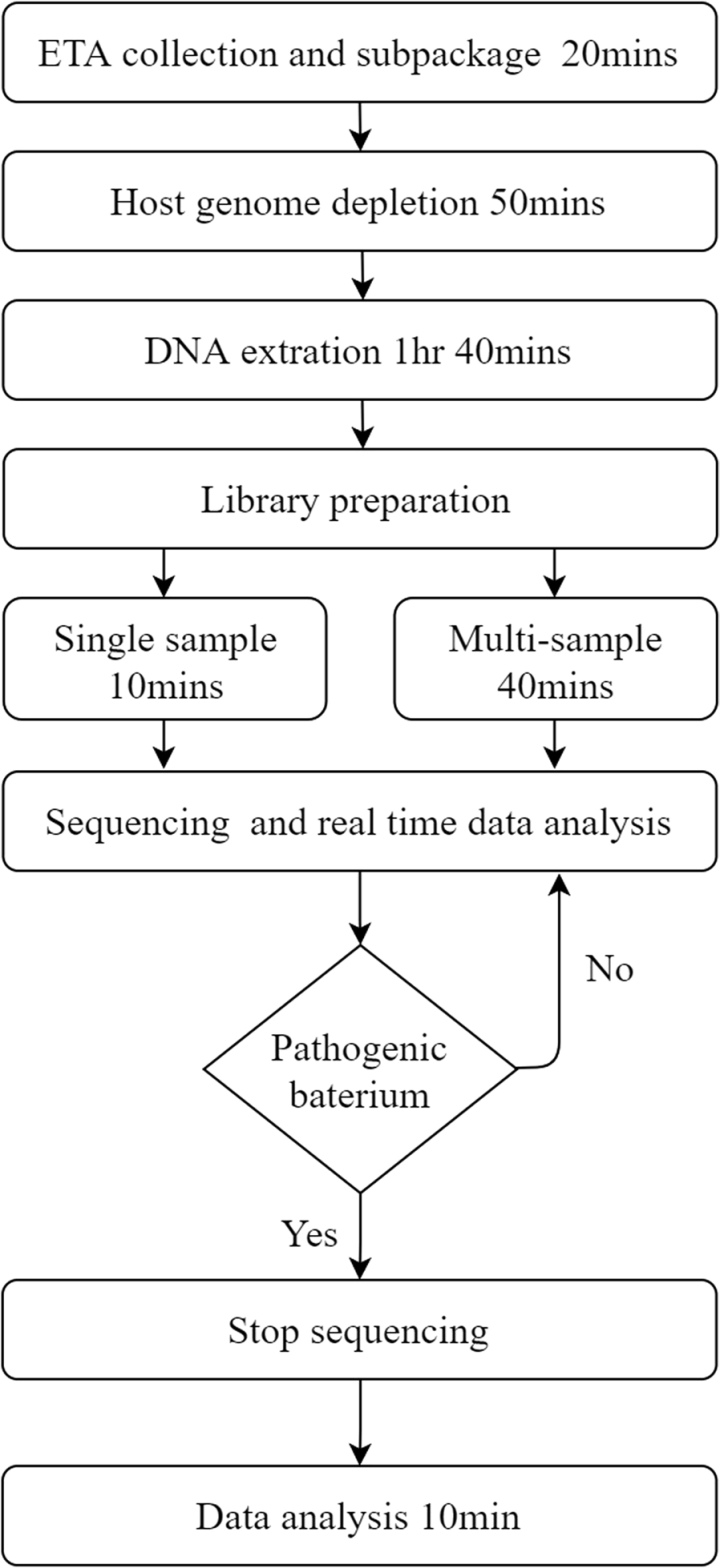
Sample processing and analysis pipeline

The undepleted DNA library construction and depleted DNA non-PCR-amplified library construction were performed using a rapid sequencing kit (SQK-RAD004, ONT, Oxford, UK) and rapid barcode kit (SQK-RBK004, ONT, Oxford, UK), while the depleted DNA PCR- amplified library construction was performed using a rapid PCR barcode kit (SQK-RPB004, ONT, Oxford, UK). The Non-PCR-amplified library construction method was performed following the instructions, and 400 ng of DNA from each sample was used (when the maximum amount of 7.5 μl of DNA was less than 400 ng, then 7.5 μl was used) for sequencing. The PCR amplification library construction method was carried out according to the instructions. 5 ng of DNA was used for each sample, the extension time was shortened from 6 to 4 min, and the amplification cycle was increased from 14 to 25 cycles [18]. Sequencing was performed using MinION (ONT, Oxford, UK) and R9.4 flowcellls (FLO-MIN106D, ONT, Oxford, UK). Raw data collection and base-calling were performed using MinKNOW (v.19.12.5, ONT, Oxford, UK) and Guppy (v.3.2.10, ONT, Oxford, UK) software. The data were collected and analysed in real time. Sequencing was continued for 1–2 h after the pathogens may cause VAP (the pathogens leading to VAP was determined according to the previous research results and Chinese VAP Guideline [7, 18, 19] and the isolation reports of VAP infection pathogens in the PUTH, the possible pathogens of VAP were determined by clinicians from Respiratory Department of PUTH) were identified. If no more pathogenic bacteria were detected, sequencing was stopped (Figs. 3 and 4).

The raw data generated by sequencing were filtered using NanoFilt (v.2.7.1) for joint sequence resection (–headcrop 150 –tailcrop 50) and low-quality segments (-q 7 -l 500) and NanoPlot (v.1.32.1) for filtration quality statistics and visualization. Minimap2 (v.2.17) was used to align the filtered clean FASTQ file with the Human GRCh38 Genome (NCBI). SamTools (v.1.11) was used to extract the unaligned sequence (-f 4) and to convert the generated data to FASTQ format. Kraken2 (v.2.1.1) was used for sequence classification (Fig. 3).

### qRT-PCR

qRT-PCR was used to confirm 7 identified pathogens in this study. In each sample, 10 μl of SYBR Master Mix (11184ES08, Yasen, Shanghai, China), 7.2 μl of NF-water, 0.4 μl of forward and reverse primer (synthesized by Beijing Ruibio Biotech Co., Ltd) (Additional file 1: Table S1), and 2 μl of DNA samples were added. Bacteria from ATCC were extracted as a positive control group of pathogens (Additional file 1: Table S1). DNA of the A549 cell line was extracted and used as a human genomic positive control group. The PCR cycling conditions were set as pre-incubation at 95 °C for 2 min; amplification for 40 cycles at 95 °C for 10 s and 60 °C for 30 s; and the final melting curve was 95 °C for 15 s, 60 °C for 60 s, and 95 °C for 15 s. The results were analysed using CT values.

### Statistical analysis

The qRT-PCR results were analysed using a T test, and the sensitivity and specificity used a binomial distribution. P value less than 0.05 was considered to indicate a significant difference. R (v.4.0.3) and SPSS (v.19) were used for statistical analysis, and the tool http://vassarstats.net/ was used for the sensitivity and specificity calculation. The images were produced using OriginPro 2017C (b8.4.2.380), Microsoft office PowerPoint 2019, R and Adobe Photoshop CC 2018.

### Data availability

All clinical samples metagenomic sequencing datum are available via China National Center for Bioinformation (www.cncb.ac.cn) under Project PRJCA006892.

## Results

### Host genome depletion

**Fig. 5:**
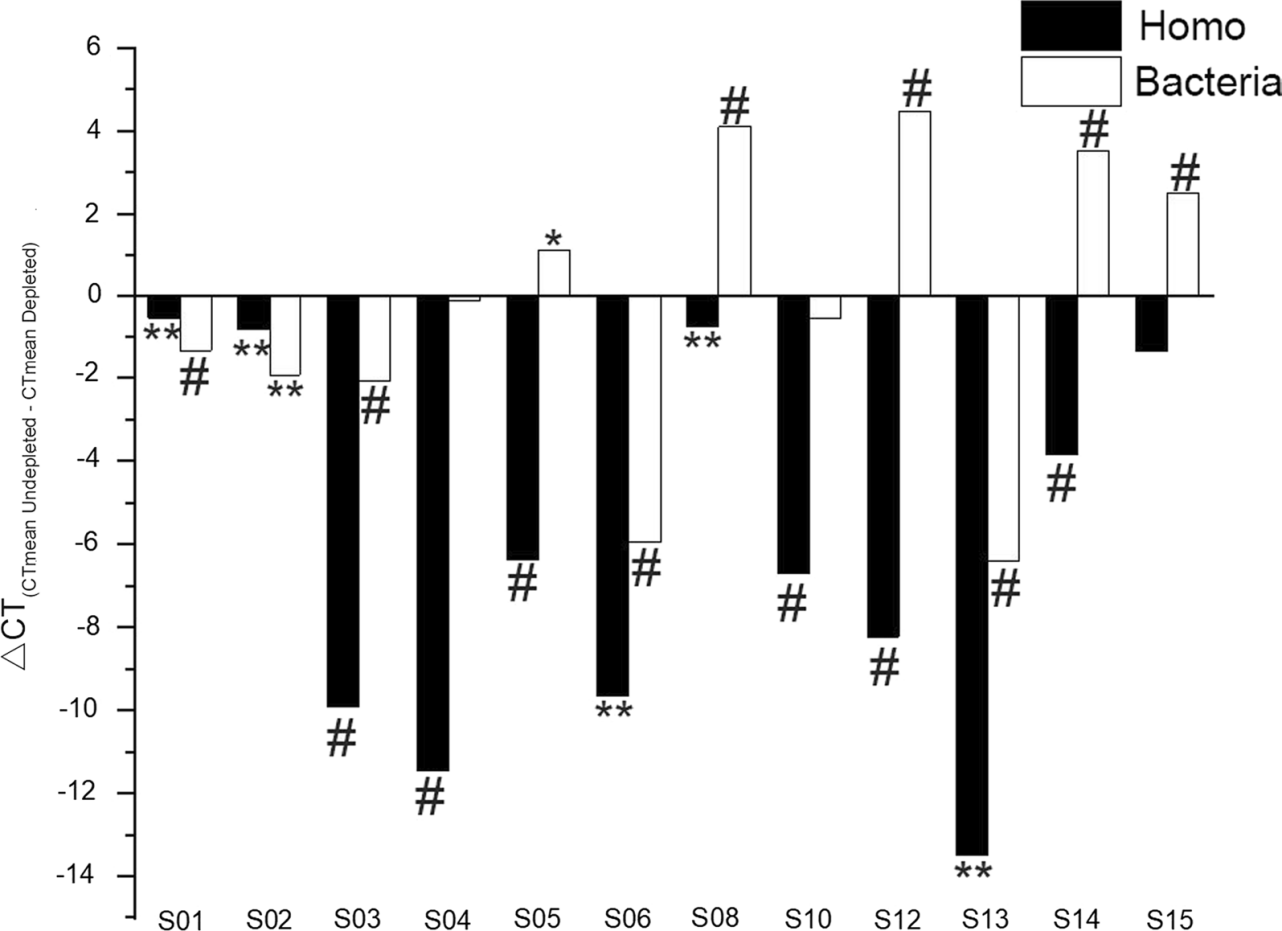
The influence of the host genome depletion procedure on the Homo and bacterial genomes. qRT-PCR was used to detect the CT values of the Homo and the bacterial genomes. △CT over 0 indicates that the genome content is higher in the depleted group, and △CT less than 0 indicates that the genome content is higher in the undepleted group. *: P < 0.05; **: P < 0.01; #: P < 0.001

12 samples with enough volume were used to evaluate the efficiency of host genome depletion in qRT-PCR method. According to Table 1 and Fig. 5, part of the human genome was successfully removed from all of the samples, and 11 of them showed significant differences in the content of the host genome between the depleted group and the undepleted group.

**Table 1:** Human depletion effect

Sample ID	Treatment	Human DNA assay (Ct mean ± SD)	Human depletion [△Ct (CT mean_Undepleted_−CT mean_Depleted_)]	Human depletion (P value)	Bacteria DNA assay (Ct mean ± SD)	Bacteria concentration change [△Ct (CT mean_Undepleted_−CT mean_Depleted_)]	Bacteria concentration change (P value)
S01	Depleted Undepleted	18.568 ± 0.160 18.059 ± 0.084	−0.51	0.004	21.509 ± 0.055 20.186 ± 0.060	−1.32	< 0.001
S02	Depleted Undepleted	19.629 ± 0.049 18.876 ± 0.138	−0.82	0.005	20.693 ± 0.021 18.784 ± 0.128	−1.91	0.001
S03	Depleted Undepleted	28.750 ± 0.202 18.843 ± 0.050	−9.9	< 0.001	25.248 ± 0.211 23.190 ± .0111	−2.05	< 0.001
S04	Depleted Undepleted	30.507 ± 0.511 19.077 ± 0.067	−11.43	< 0.001	19.405 ± 0.169 19.323 ± 0.015	−0.08	0.486
S05	Depleted Undepleted	24.546 ± 0.084 18.197 ± 0.386	−6.35	< 0.001	19.693 ± 0.169 20.827 ± 0.308	−1.13	0.017
S06	Depleted Undepleted	28.146 ± 1.059 18.495 ± 0.403	−9.65	0.002	26.523 ± 0.144 20.589 ± 0.204	−5.93	< 0.001
S08	Depleted Undepleted	19.629 ± 0.086 18.876 ± 0.179	−0.75	0.001	21.538 ± 0.053 25.652 ± 0.222	4.11	< 0.001
S10	Depleted Undepleted	25.587 ± 0.093 18.903 ± 0.421	−6.68	< 0.001	20.115 ± 0.286 19.610 ± 0.164	−0.504	0.072
S12	Depleted Undepleted	27.051 ± 0.199 18.835 ± 0.129	−8.21	< 0.001	24.641 ± 0.077 29.123 ± 0.185	4.48	< 0.001
S13	Depleted Undepleted	31.984 ± 1.372 18.505 ± 0.143	−13.48	0.003	29.092 ± 0.055 22.696 ± 0.150	−6.40	< 0.001
S14	Depleted Undepleted	23.710 ± 0.319 19.869 ± 0.131	−3.84	< 0.001	13.605 ± 0.084 17.143 ± 0.067	3.53	< 0.001
S15	Depleted Undepleted	20.807 ± 0.838 19.466 ± 0.282	−1.34	0.097	14.208 ± 0.175 16.705 ± 0.193	2.50	< 0.001

**Fig. 6:**
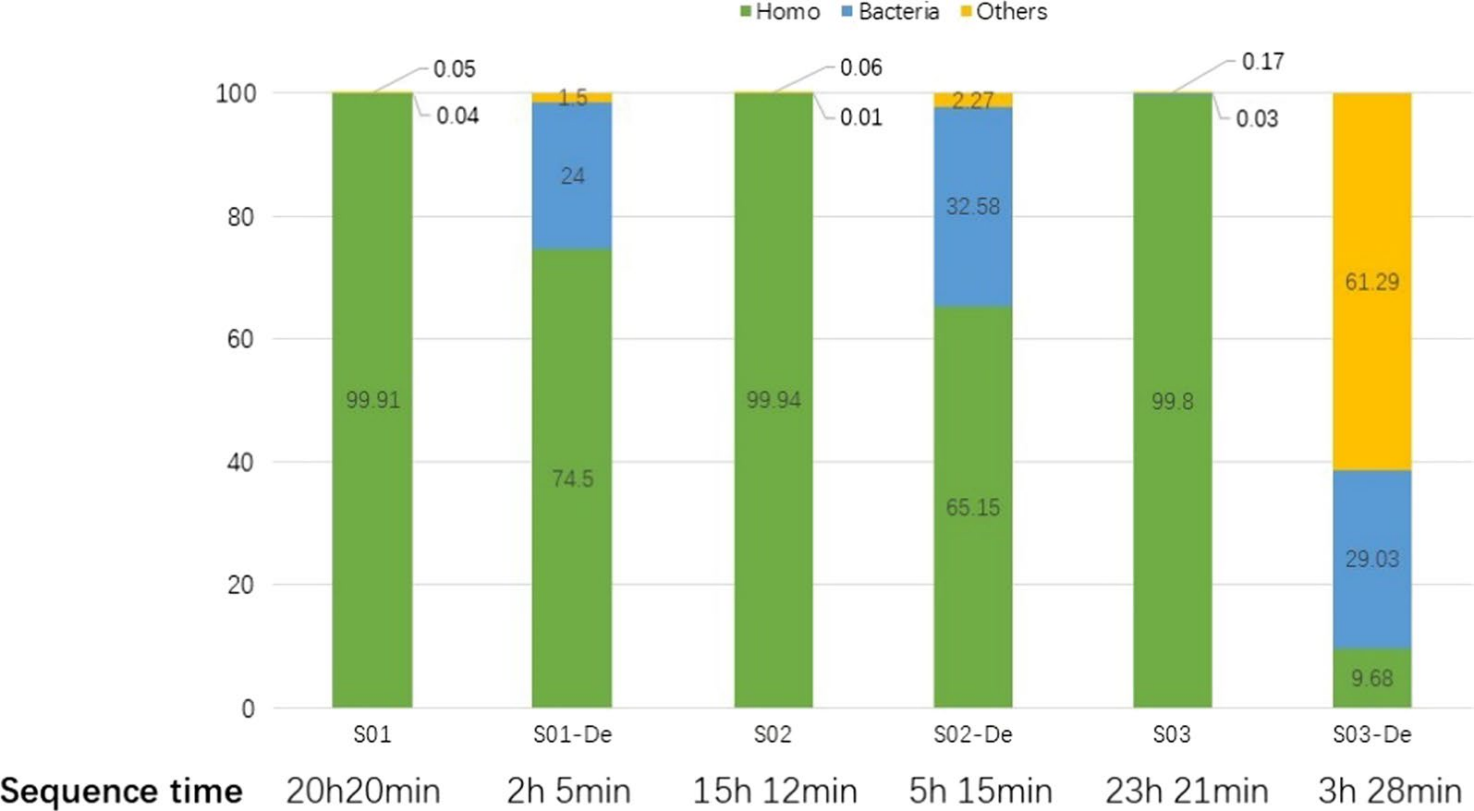
Comparison of single-sample sequencing with or without host genome depletion. Samples S01, S02 and S03 underwent single-sample sequencing using new flow cells each time to compare the difference between the host genome-depleted group and the undepleted group. S01, S02 and S03 represent samples without host genome depletion; S01-De, S02-De and S03-De represent samples with host genome depletion

3 of the 12 ETA samples (S01, S02 and S03) were used to compare the efficacy in metagenomic sequencing method with or without host genome depletion. As indicated in Fig. 6, for undepleted samples, the results were demined by the Homo genome, the bacterial genome accounted for only 0.01–0.04%, and no clear pathogen causing VAP was found after sequencing for 15–23 h. The same sequence process was performed on these 3 patients (S01, S02 and S03) after saponin depletion. After sequencing for 2–5 h, the percentage of bacteria was clearly increased, and pathogen detection results consistent with the clinical culture results were obtained (Fig. 6, Additional file 2: Table S2), suggesting that the sequencing efficiency can be greatly improved by depletion.

However, whether saponins can also deplete bacteria is not clear. After the saponin depletion procedure, the bacterial DNA content of 5 samples was significantly increased (S05, S08, S12, S14, and S15), 5 were significantly decreased (S01, S02, S03, S06, and S13), and 2 showed no significant difference (S04 and S10) (Table 1, Figure 5). To explore whether saponin depletion progress could also deplete bacterial genomes, 7 cultured pathogens obtained from ATCC and the clinical culture department of PUTH were used and equally divided into two parts, following the comparison procedure in Fig. 2. As shown in Table 2, saponin depletion had no significant effect on the pathogens except *S. pneumoniae*. The concentration of the *S. pneumoniae* strain from ATCC decreased by approximately 0.31 times after depletion, while the clinical strains decreased by approximately 0.23 times.

**Table 2:** Influence of human depletion on pathogens

Pathogen	Source	Depleted (CT mean ± SD)	Undepleted (CT mean ± SD)	P value
*A. baumannii*	ATCC BAA-747	14.388 ± 0.179	14.021 ± 0.143	0.053
	Clinical isolation	15.908 ± 0.504	15.024 ± 0.182	0.080
*P. aeruginosa*	ATCC 27853	13.172 ± 0.035	13.136 ± 0.065	0.455
	Clinical isolation	12.706 ± 0.041	12.615 ± 0.118	0.302
*K. pneumoniae*	ATCC 700603	18.995 ± 0.509	18.794 ± 0.227	0.580
	Clinical isolation	18.756 ± 0.546	17.828 ± 0.398	0.082
*S. aureus*	ATCC 29213	18.712 ± 0.070	18.517 ± 0.107	0.067
	Clinical isolation	17.487 ± 0.137	17.302 ± 0.190	0.249
*S. maltophilia*	ATCC 17666	11.955 ± 0.163	12.003 ± 0.204	0.767
	Clinical isolation	11.146 ± 0.065	11.094 ± 0.042	0.313
*S. pneumoniae*	ATCC 49619	25.392 ± 0.095	17.529 ± 0.321	< 0.001
	Clinical isolation	22.525 ± 0.067	17.243 ± 0.081	< 0.001
*E. coli*	ATCC 25922	12.357 ± 0.121	12.104 ± 0.081	0.096
	Clinical isolation	13.948 ± 0.112	13.808 ± 0.425	0.630

### Library construction

ONT provides two library preparations methods: that differ in whether PCR amplification is performed before library construction. Our laboratory compared the performance of the 2 methods in terms of sequencing duration and sequencing results. As described in Table 3, in the 29 sequencing results, the non-PCR amplification method and the PCR amplification method had similar sensitivity, while the specificity of the non-PCR amplification method was better than that of the PCR amplification method (average sensitivity: 85.8% vs. 86.35%, average specificity: 100% vs. 91.15%) (detailed data in Additional file 3: Table S3). In addition, the average sequencing time per sample for both methods were similar, but the PCR amplification method required an additional 2 h and 16 min of amplification, so the non-PCR amplification method took less time overall. Based on the sequencing duration and performance results, the non-PCR amplification method can be used as the first choice for sequencing.

**Table 3:** Sensitivity and specificity of non-PCR amplified sequencing and PCR amplified sequencing

Pathogen	Sensitivity (95% CI)			Specificity (95% CI)		
	Non-PCR amplified sequencing	PCR amplified sequencing	qRT-PCR	Non-PCR amplified sequencing	PCR amplified sequencing	qRT-PCR
*A. baumannii*	87.5% (60.41–97.8%)	93.75% (67.71–99.67%)	100% (75.93–100%)	100% (71.66–100%)	84.62% (53.66–97.29%)	100% (71.66–100%)
*P. aeruginosa*	66.67% (24.11–94%)	50% (13.95–86.05%)	100% (51.68–100%)	100% (82.19–100%)	100% (82.19–100%)	100% (82.19–100%)
*K. pneumoniae*	71.43% (30.26–94.89%)	85.71% (42.01–99.25%)	57.14% (20.24–88.19%)	100% (81.5–100%)	81.82% (58.99–94.01%)	100% (81.50–100%)
*S. aureus*	75% (35.58–95.55%)	75% (35.58–95.55%)	87.5% (46.68–99.34%)	100% (80.76–100%)	100% (80.76–100%)	100% (80.76–100%)
*S. maltophilia*	100% (31–100%)	100% (31–100%)	100% (31–100%)	100% (83.98–100%)	100% (83.98–100%)	100% (83.98–100%)
*S. pneumoniae*	100% (39.58–100%)	100% (39.58–100%)	100% (39.58–100%)	100% (83.42–100%)	92% (72.5–98.6%)	100% (83.42–100%)
*E. coli*	100% (5.46–100%)	100% (5.46–100%)	100% (5.46–100%)	100% (84.98–100%)	82.14% (62.42–93.23%)	96.43% (79.76–100%)

### Data analysis

**Fig. 7:**
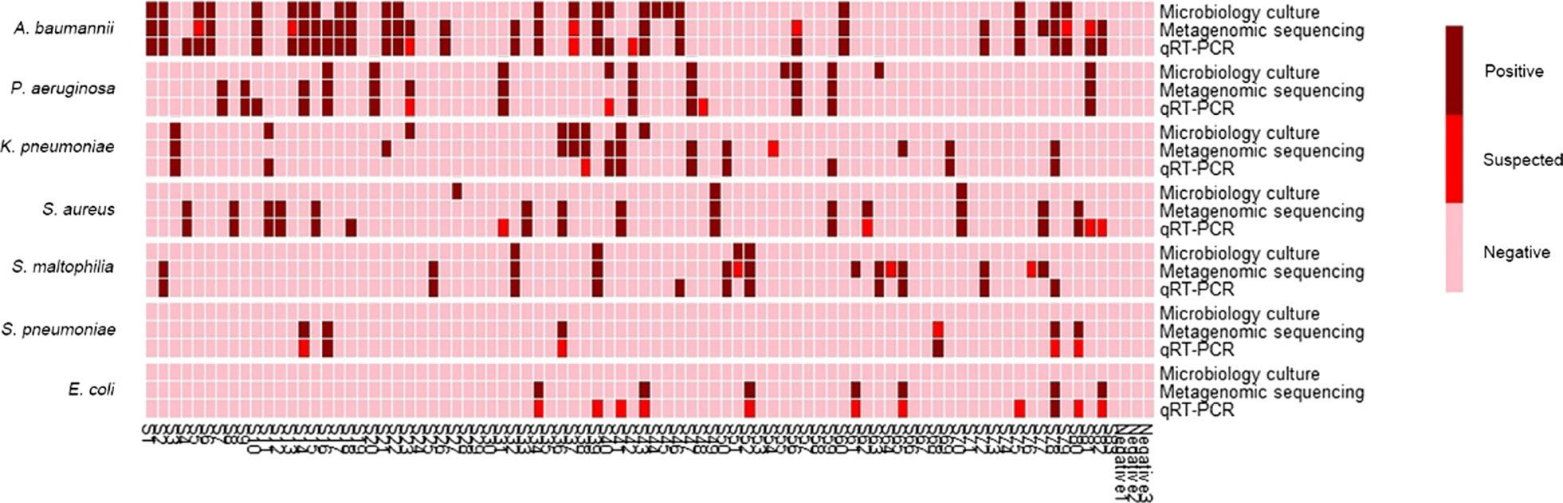
Comparison of microbiological culture, metagenomic sequencing and qRT-PCR. Positive: 1). Microbiology culture: microbiology results report positive; 2). Metagenomic sequencing: reads count over 1 and 1% of all pathogen genomes; 3). qRT-PCR: CT value < 30 and significantly less than negative control; Suspected: 1). Metagenomic sequencing: only has 1 read but over 10% of all genomes; 2). qRT-PCR: CT value between 30–35 and significantly less than negative control; Negative: others. Meeting one of the following conditions indicates that pathogens exist: 1). Microbiology culture positive; 2). qRT-PCR positive; 3). qRT-PCR suspected with metagenomic sequencing positive or suspected

DNA extraction and sequencing identification of 83 samples were conducted according to Fig. 4, and the whole procedure took 5–6 h from the time of ETA extraction to the obtainment of classification results. The identification results are shown in Fig. 7 and Additional file 2: Table S2. As indicated in Table 4, the average sensitivity of metagenomic sequencing was much better than that of clinical culture but very close to that of qRT-PCR (clinical culture 37.77% vs. metagenomic sequencing 89.15% vs. qRT-PCR 90.29%); the average specificity of metagenomic sequencing was the best among all methods, while that of qRT-PCR was the lowest (clinical culture 98.62% vs. metagenomic sequencing 98.8% vs. qRT-PCR 97.71%).

**Table 4:** Sensitivity and specificity of microbiology culture, metagenomic sequencing and qRT-PCR

Pathogen	Sensitivity (95%CI)			Specificity (95%CI)		
	Microbiology culture	Metagenomic sequencing	qRT-PCR	Microbiology culture	Metagenomic sequencing	qRT-PCR
*A. baumannii*	73.53% (55.35–86.49%)	88.23% (71.61–96.16%)	94.12% (78.94–98.97%)	100% (90.94–100%)	100% (90.94–100%)	97.96% (87.76–99.89%)
*P. aeruginosa*	68.75% (41.48–87.87%)	75% (47.41–91.67%)	87.5% (60.41–97.80%)	100% (93.24–100%)	100% (93.24–100%)	98.51% (90.86–99.92%)
*K. pneumoniae*	53.33% (27.42–77.72%)	73.33% (44.83–91.09%)	66.67% (38.69–87.01%)	90.36% (81.39–95.45%)	97.06% (88.84–99.49%)	100% (93.34–100%)
*S. aureus*	18.75% (4.97–46.31%)	87.5% (60.41–97.8%)	93.75% (67.71–99.67%)	100% (93.24–100%)	100% (93.24–100%)	95.52% (86.63–98.84%)
*S. maltophilia*	50% (20.14–79.86%)	100% (65.55–100%)	90% (54.12–99.48%)	100% (93.77–100%)	94.52% (85.84–98.23%)	97.26% (89.56–99.52%)
*S. pneumoniae*	0% (0–53.71%)	100% (51.68–100%)	100% (51.68–100%)	100% (94.15–100%)	100% (94.08–100%)	100% (94.08–100%)
*E. coli*	0% (0–43.9%)	100% (56.09–100%)	100% (56.09–100%)	100% (94–100%)	100% (94–100%)	94.74% (86.36–98.3%)

## Discussion

Pathogen identification is crucial in VAP diagnosis and treatment, and building a time-saving method that is easy to use in clinical departments could provide a guidance for clinical antibiotic management, reduce the empiric antibiotic therapy duration, narrow the antibiotic and reduce the chance of bacteria resistance and useless antibiotic exposure. Nanopore technology has been used in the diagnosis of several epidemiological cases [13, 17, 18, 20–24]. However, there are no unified procedures for addressing respiratory samples, and the efficiency of different methods for treating different pathogens is not clear. Here, we compared different methods and provided a theoretical basis for the choice of methodology, providing a newly rapid pathogen identification method.

This study included 83 ETA samples from patients with suspected VAP who had been intubated for more than 48 h. The host genome, which is 10^5^ times more abundant than the bacterial genome, could cover up pathogen information during metagenomic sequencing [18, 25]. Saponin, as a detergent, breaks the cytomembrane of wall-less host cells without influencing the bacterial cytoderm. The cell-free DNA released by broken host cells can be digested by DNA digesting enzymes to reduce the concentration of the host genome [18]. As previously indicated by other researchers [18, 26], our experiment also found that DNA extracted directly from ETA was dominated by the host genome, leading to poor sequencing performance with no pathogen detected. Therefore, the removal of the host genome has become a necessary step in sample processing. Host genomes were removed from all 12 samples after depletion, and the genomic abundance of pathogens and the sequence time were significantly improved after depletion, suggesting that the depletion operation of saponins was of great significance for the optimization of sequencing results.

However, there is no clear conclusion regarding the effect of saponin depletion on the pathogen genome. After comparing the effects of depletion on the 7 ATCC acquired and clinically cultured pathogens involved in this study, the results showed that depletion did not significantly affect the abundance of 6 pathogens but did affect *S. pneumoniae*. This result is similar to the research result of Charalampous et al. from the UK, which may be due to the simultaneous lysis of *S. pneumoniae* genes during the lysis process of the human genome [18, 27]. In this study, however, among 83 cases, 6 cases involving *S. pneumoniae* (S14, S16, S36, S68, S78, and S80) were negative by clinical microbiology culture but positive according to the metagenomic sequencing results, and all 6 samples also suggested the existence of *S. pneumoniae* from the qRT-PCR results. Although DNA extraction from human sources may damage pathogens, sequencing is still a better choice for the detection of *S. pneumoniae*.

Whether to conduct PCR amplification during the library building process is also one of the issues that needs to be discussed. One of the advantages of Nanopore sequencing is that DNA sequence information can be obtained without PCR amplification, thus preserving the methylation and other modification information on the DNA, which is conducive to further data mining and processing. Moreover, building libraries without PCR amplification could reduce the augmented error and save time by forgoing the amplification. In this study, the differences in pathogen detection between PCR amplification and non-PCR amplification in 29 samples were compared, and for most pathogens, adequate and effective pathogen information could still be obtained without PCR amplification before sequencing.

Some samples showed positive culture and qRT-PCR results but negative sequencing results (*K. pneumoniae*: S11; *P. aeruginosa,* and *A. baumannii*: S40), which may be due to the high content of host or oropharyngeal pathogen genome content that covered up the pathogen information (about 55% of S11 were comprised by oropharyngeal pathogen and homo sapiens, and 80% of S40 were comprised by oropharyngeal pathogen), and the DNA quality was low in those samples (both DNA quality were beyond 1.8–2.0 of OD 260/280 or 260/230). As Nanopore sequencing has a high DNA integrity requirement for the fragments, low-quality DNA may not be successfully read. Proper protection and cleaning of the oropharynx during sampling and adequate saponin mixing of samples could reduce the concentration of host and oropharyngeal pathogen genomes [18]. For DNA samples with low quality, it is better to re-extract DNA from ETAs to get DNA with good quality, if possible, as the sensitivity of low-quality DNA sequencing results is lower than that of full-quality DNA sequencing results (Table 3 row non-PCR-amplification and Table 4 row metagenomic sequencing).

Some samples showed positive clinical culture results but negative results by sequencing and qRT-PCR (*A. baumannii*: S44, S45; *P. aeruginosa*: S55, S63; *and K. pneumoniae*: S23, S43), which may be due to sampling error. A study by Dickson RP et al. found that the bacterial flora distribution differs from the oral cavity to the lower lung lobes in the human respiratory tract [28], and the collection of ETAs is a blind process, so there is the possibility that the sample was taken from different parts of the lower respiratory tract. Repeated sampling may appropriately avoid the occurrence of such phenomena, and this is in need of further study.

Our study utilized a new method for clinicians to identify bacteria in the lower respiratory tract from suspected VAP patients. However, this method has some limitations that need to be further studied. First, the host genome depletion process could affect different pathogens to different degrees, and the balancing of host genome depletion and pathogen genome protection still needs further work. Whether the pathogenic bacteria were depleted or covered up by abundant species groups or actually did not exist, further study is needed. Second, the definition of Positive and Negative in metagenomic sequencing results is also another issue need to be discussed. In this study, we modified the criteria for several times, and found that the definition of “pathogen reads over 1 read and 1% of all pathogenic genome” to define positive and “pathogen only have 1 read but over 10% of all genomics” to define suspect had best sensitivity and specificity. But this definition is only used in this study, if this is proper for all metagenomic sequencing results still need further research. Third, for patients with tracheal intubation for 48 h or more, the types of pathogens in the lower respiratory tract decreased with the extension of intubation time, but the abundance of individual pathogens increased with the extension of intubation time [29]. For patients with newly intubated trachea and suspected VAP, the sequencing results often present a mixed form of multiple pathogens. In addition, the number of reads of different pathogens may vary greatly within the sequencing results of the same sample. For such samples, it is still necessary for clinicians to make judgements about the specific pathogenic bacterial types and precise drug use in combination with the clinical manifestations of patients.

## Supplementary Information

**Additional file 1: **qRT-PCR gene targets and primer sequences.

**Additional file 2:** Results Comparation.

**Additional file 3**: Comparation of PCR amplificated sequencing and non-PCR amplificated sequencing.

## Data Availability

The datasets used and/or analysed during the current study are available from the corresponding author on reasonable request.

## Abbreviations

VAP: Ventilator-associated pneumonia
ETA: Endotracheal aspirate
MV: Mechanical ventilation
PUTH: Peking University Third Hospital
NF-water: Nuclease-free water
McF: McFarland
ATCC: American Type Culture Collection
HL-SAN: Heat-labile salt active nuclease
*P. aeruginosa*: *Pseudomonas aeruginosa*
*A. baumannii*: *Acinetobacter baumannii*
*K. pneumoniae*: *Klebsiella pneumoniae*
*S. aureus*: *Staphylococcus aureus*
*S. maltophilia*: *Stenotrophomonas maltophilia*
*S. pneumoniae*: *Streptococcus pneumoniae*
*E. coli*: *Escherichia coli*
