# Supplementary material for: Rapid identification of pathogens associated with ventilator-associated pneumonia by Nanopore sequencing
Source: Respir Res. 2021 Dec 10;22:310. doi: 10.1186/s12931-021-01909-3 (PMC8665642; doi:10.1186/s12931-021-01909-3)
Supplement: Supplementary file 1 — Additional file 1: qRT-PCR gene targets and primer sequences. [file 12931_2021_1909_MOESM1_ESM.docx]

**Additional File 1 qRT-PCR gene targets and primer sequences**

| **Organism** | **Target gene** | **Forward primer (5’-3’)** | **Reverse primer**  **(5’-3’)** | **Positive control** | **Reference** |
| --- | --- | --- | --- | --- | --- |
| Human | RNA  polymeraseA | TGAAGCCGTGCGGAAGG | ACAAGAGAGCCAAGTGTCG | A549 | (17) |
| Bacteria | V3-V4 | TCGTCGGCAGCGTCAGATGTGTATAAGAGACAGCCTACGGGNGGCWGCAG | GTCTCGTGGGCTCGGAGATGTGTATAAGAGACAGGACTACHVGGGTATCTAATCC | ATCC 700603 | (17) |
| *A. baumannii* | K09-14 | CACGCCGTAAGAGTGCATTA | AACGGAGCTTGTCAGGGTTA | ATCC BAA-747 | Designed by company |
| *P. aeruginosa* | porL | AGCCTTCCTGGTCCCCTTAC | CCTAATGAACCCCAGTGTATAAGTTTG | ATCC 27853 | (17) |
| *K. pneumoniae* | khe | TGATTGCATTCGCCACTGG | GGTCAACCCAACGATCCTG | ATCC 700603 | (27) |
| *S. aureus* | nuc | TTGTAGTTTCAAGTCTAAGTAGCTCAGC | TTGCACTATATACTGTTGGATCTTCAG | ATCC 29213 | Designed by company |
| *S. maltophilia* | 23S rRNA | GCCGAAAGCCCAAGGTTT | CGACTTTCGTCCTCGCCTTA | ATCC 17666 | (17) |
| *S. pneumoniae* | ply | AGCGATAGCTTTCTCCAAGTGG | CTTAGCCAACAAATCGTTTACCG | ATCC 49619 | (26) |
| *E. coli* | cyaA | CGATAATCGCCAGATGGC | CCTAAGTTGCAGGAGATGG | ATCC 25922 | (17) |
