# Supplementary material for: Rapid identification of pathogens associated with ventilator-associated pneumonia by Nanopore sequencing
Source: Respir Res. 2021 Dec 10;22:310. doi: 10.1186/s12931-021-01909-3 (PMC8665642; doi:10.1186/s12931-021-01909-3)
Supplement: Supplementary file 2 — Additional file 2: Results Comparation. [file 12931_2021_1909_MOESM2_ESM.docx]

**Additional File 2 Results Comparation (part 1)**

| **Sample ID** | **Microbiology culture results** | **Sequencing results (sorted by reads count)** | **PCR results** |
| --- | --- | --- | --- |
| S01 | *A. baumannii 4+* | *A. baumannii* | *A. baumannii* |
| S02 | *A. baumannii 3+* | *S. maltophilia*  *A. baumannii* | *S. maltophilia*  *A. baumannii* |
| S03 | *K. pneumoniae 4+* | *K. pneumoniae* | *K. pneumoniae* |
| S04 | None | *S. aureus* | *S. aureus*  *A. baumannii* |
| S05 | *A. baumannii 2+* | *A. baumannii* | *A. baumannii* |
| S06 | *A. baumannii 4+* | *A. baumannii* | *A. baumannii* |
| S07 | None | *P. aeruginosa* | *P. aeruginosa* |
| S08 | None | *S. aureus* | *S. aureus* |
| S09 | None | *P. aeruginosa* | *P. aeruginosa* |
| S10 | *A. baumannii 4+* | *A. baumannii* | *A. baumannii*  *P. aeruginosa* |
| S11 | *K. pneumoniae 4+* | *S. aureus* | *K. pneumoniae*  *S. aureus* |
| S12 | None | *S. aureus* | *S. aureus* |
| S13 | *A. baumannii 3+* | *A. baumannii* | *A. baumannii* |
| S14 | *A. baumannii 3+* | *P. aeruginosa*  *A. baumannii*  *S. pneumoniae* | *P. aeruginosa*  *A. baumannii*  *S. pneumoniae** |
| S15 | *A. baumannii 3+* | *S. aureus*  *A. baumannii* | *S. aureus*  *A. baumannii* |
| S16 | *P. aeruginosa 3+* | *P. aeruginosa*  *S. pneumoniae*  *A. baumannii* | *P. aeruginosa*  *S. pneumoniae*  *A. baumannii* |
| S17 | *A. baumannii 2+* | *A. baumannii* | *A. baumannii* |
| S18 | *A. baumannii 4+* | *A. baumannii* | *A. baumannii*  *S. aureus* |
| S19 | None | None | None |
| S20 | *P. aeruginosa 2+* | *P. aeruginosa* | *P. aeruginosa* |
| S21 | *A. baumannii 3+* | *A. baumannii*  *K. pneumoniae* | *A. baumannii* |
| S22 | *A. baumannii 2+* | *A. baumannii* | *A. baumannii* |
| S23 | *K. pneumoniae 3+* | *P. aeruginosa*  *A. baumannii* | *P. aeruginosa*  *A. baumannii** |
| S24 | None | None | None |
| S25 | *S. maltophilia 4+* | *S. maltophilia* | *S. maltophilia* |

**Additional File 2 Results Comparation (part 2)**

| **Sample ID** | **Microbiology culture results** | **Sequencing results (sorted by reads count)** | **PCR results** |
| --- | --- | --- | --- |
| S26 | *A. baumannii 3+* | *A. baumannii* | *A. baumannii* |
| S27 | *S. aureus 2+* | None | None |
| S28 | None | None | None |
| S29 | None | None | None |
| S30 | None | None | None |
| S31 | *P. aeruginosa 3+* | *P. aeruginosa* | *P. aeruginosa*  *S. aureus* |
| S32 | *S. maltophilia 4+* | *A. baumannii*  *S. maltophilia* | *A. baumannii*  *S. maltophilia* |
| S33 | None | *S. aureus* | *S. aureus* |
| S34 | *A. baumannii 4+* | *A. baumannii*  *E. coli* | *A. baumannii*  *E. coli* |
| S35 | None | None | None |
| S36 | *K. pneumoniae 1+* | *S. aureus*  *S. pneumoniae*  *K. pneumoniae* | *S. aureus*  *S. pneumoniae* |
| S37 | *K. pneumoniae 4+*  *A. baumannii 4+* | *K. pneumoniae*  *A. baumannii* | *A. baumannii* |
| S38 | *K. pneumoniae 3+* | *K. pneumoniae* | *K. pneumoniae* |
| S39 | *A. baumannii 4+*  *S. maltophilia 4+* | *A. baumannii*  *S. maltophilia* | *A. baumannii*  *S. maltophilia*  *E. coli* |
| S40 | *P. aeruginosa 3+*  *A. baumannii 2+* | *K. pneumoniae* | *P. aeruginosa*  *A. baumannii*  *K. pneumoniae* |
| S41 | *K. pneumoniae 3+* | *K. pneumoniae*  *S. aureus* | *K. pneumoniae*  *S. aureus*  *E. coli* |
| S42 | *P. aeruginosa 3+* | *P. aeruginosa* | *P. aeruginosa*  *A. baumannii* |
| S43 | *A. baumannii 3+*  *K. pneumoniae 3+* | *A. baumannii*  *E. coli* | *A. baumannii*  *E. coli** |
| S44 | *A. baumannii 2+* | None | None |
| S45 | *A. baumannii 2+* | None | None |
| S46 | *A. baumannii 2+* | *A. baumannii* | *A. baumannii*  *S. maltophilia* |

**Additional File 2 Results Comparation (part 3)**

| **Sample ID** | **Microbiology culture results** | **Sequencing results (sorted by reads count)** | **PCR results** |
| --- | --- | --- | --- |
| S47 | *P. aeruginosa 3+* | *P. aeruginosa*  *K. pneumoniae* | *P. aeruginosa*  *K. pneumoniae* |
| S48 | None | None | *P. aeruginosa* |
| S49 | *S. aureus 2+* | *S. aureus* | *S. aureus* |
| S50 | None | *S. maltophilia*  *K. pneumoniae* | *S. maltophilia*  *K. pneumoniae* |
| S51 | *S. maltophilia 2+* | *S. maltophilia^#^* | None |
| S52 | *S. maltophilia 4+* | *S. maltophilia*  *E. coli* | *S. maltophilia*  *E. coli* |
| S53 | None | None | None |
| S54 | None | *K. pneumoniae* | None |
| S55 | *P. aeruginosa 3+* | None | None |
| S56 | *P. aeruginosa 2+* | *P. aeruginosa*  *A. baumannii* | *P. aeruginosa*  *A. baumannii* |
| S57 | None | None | None |
| S58 | None | None | None |
| S59 | *P. aeruginosa 2+* | *S. aureus*  *P. aeruginosa* | *S. aureus*  *P. aeruginosa*  *K. pneumoniae* |
| S60 | *A. baumannii 2+* | *A. baumannii* | *A. baumannii* |
| S61 | None | *S. maltophilia*  *E. coli* | *E. coli* |
| S62 | None | *S. aureus* | *S. aureus* |
| S63 | *P. aeruginosa 2+* | *S. maltophilia* | *S. maltophilia* |
| S64 | None | *S. maltophilia^#^* | None |
| S65 | *K. pneumoniae 2+* | *S. maltophilia*  *E. coli*  *K. pneumoniae* | *S. maltophilia*  *E. coli* |
| S66 | None | None | None |
| S67 | None | None | None |
| S68 | None | *S. pneumoniae* | *S. pneumoniae* |
| S69 | None | *K. pneumoniae* | *K. pneumoniae* |
| S70 | *S. aureus 3+* | *S. aureus* | *S. aureus* |
| S71 | None | None | None |

**Additional File 2 Results Comparation (part 4)**

| **Sample ID** | **Microbiology culture results** | **Sequencing results (sorted by reads count)** | **PCR results** |
| --- | --- | --- | --- |
| S72 | None | *A. baumannii*  *S. maltophilia* | *A. baumannii*  *S. maltophilia* |
| S73 | None | None | None |
| S74 | None | None | None |
| S75 | *A. baumannii 4+* | *A. baumannii* | *A. baumannii*  *E. coli** |
| S76 | None | *S. maltophilia* | None |
| S77 | None | *S. aureus*  *A. baumannii*  *S. maltophilia* | *S. aureus* |
| S78 | *A. baumannii 4+* | *A. baumannii*  *K. pneumoniae*  *S. pneumoniae*  *E. coli* | *A. baumannii*  *K. pneumoniae*  *S. pneumoniae*  *E. coli*  *S. maltophilia* |
| S79 | *A. baumannii 3+* | *A. baumannii* | *A. baumannii* |
| S80 | None | *S. pneumoniae*  *S. aureus* | *S. pneumoniae*  *S. aureus*  *E. coli* |
| S81 | *P. aeruginosa 4+* | *P. aeruginosa*  *A. baumannii* | *P. aeruginosa*  *A. baumannii*  *S. aureus* |
| S82 | None | *A. baumannii*  *E. coli* | *A. baumannii*  *E. coli*  *S. aureus* |
| S83 | None | None | None |
| Negative1 | None | None | None |
| Negative2 | None | None | None |
| Negative3 | None | None | None |
