# Supplementary material for: Rapid identification of pathogens associated with ventilator-associated pneumonia by Nanopore sequencing
Source: Respir Res. 2021 Dec 10;22:310. doi: 10.1186/s12931-021-01909-3 (PMC8665642; doi:10.1186/s12931-021-01909-3)
Supplement: Supplementary file 3 — Additional file 3: Comparation of PCR amplificated sequencing and non-PCR amplificated sequencing. [file 12931_2021_1909_MOESM3_ESM.docx]

**Additional File 3 Comparation of PCR sequencing and non-PCR sequencing**

| **Sample ID** | **Microbiology culture results** | **Non-PCR sequencing** | **PCR sequencing** |
| --- | --- | --- | --- |
| S01 | *A. baumannii* | *A. baumannii* | *A. baumannii* |
| S02 | *A. baumannii* | *S. maltophilia*, A. baumannii* | *S. maltophilia*, A. baumannii, K. pneumoniae^#^* |
| S04 | None | *S. aureus** | *S. aureus*, A. baumannii** |
| S05 | *A. baumannii* | *A. baumannii* | *K. pneumoniae^#^* |
| S06 | *A. baumannii* | *A. baumannii* | *A. baumannii* |
| S10 | *A. baumannii* | *A. baumannii* | *A. baumannii* |
| S11 | *K. pneumoniae* | *S. aureus** | *S. aureus*, A. baumannii^#^, K. pneumoniae* |
| S12 | None | *S. aureus** | *S. aureus** |
| S14 | *A. baumannii* | *P. aeruginosa*, A. baumannii, S. pneumoniae** | *P. aeruginosa*, S. pneumoniae*, A. baumannii, S. maltophilia^#^, K. pneumoniae^#^* |
| S15 | *A. baumannii* | *S. aureus*, A. baumannii* | *A. baumannii, S. aureus*, E. coli^#^, S. pneumoniae^#^* |
| S16 | *P. aeruginosa* | *P. aeruginosa, S. pneumoniae^*^, A. baumannii** | *P. aeruginosa, A. baumannii*, S. pneumoniae^*^* |
| S17 | *A. baumannii* | *A. baumannii* | *A. baumannii, E. coli^#^* |
| S18 | *A. baumannii* | *A. baumannii* | *A. baumannii* |
| S19 | None | None | *E. coli^#^* |
| S20 | *P. aeruginosa* | *P. aeruginosa** | None |
| S23 | *K. pneumoniae* | *P. aeruginosa*, A. baumannii** | *A. baumannii** |
| S26 | *A. baumannii* | *A. baumannii* | *A. baumannii* |
| S27 | *S. aureus* | None | None |
| S28 | None | None | None |
| S29 | None | None | None |
| S37 | *A. baumannii, K. pneumoniae* | *K. pneumoniae, A. baumannii** | *K. pneumoniae, A. baumannii* |
| S38 | *K. pneumoniae* | *K. pneumoniae* | *K. pneumoniae* |
| S39 | *A. baumannii* | *A. baumannii, S. maltophilia** | *A. baumannii, S. maltophilia*,* |
| S40 | *P. aeruginosa, A. baumannii* | *K. pneumoniae** | *K. pneumoniae*, P. aeruginosa, E. coli^#^, S. aureus^#^, A. baumannii* |
| S41 | *K. pneumoniae* | *K. pneumoniae, S. aureus** | *K. pneumoniae, S. aureus** |
| S65 | *K. pneumoniae* | *S. maltophilia*, E. coli*, K. pneumoniae* | *E. coli*, S. maltophilia*, K. pneumoniae, A. baumannii^#^, P. aeruginosa^#^* |
| S66 | None | None | *S. pneumoniae^#^* |
| S68 | None | *S. pneumoniae** | *S. pneumoniae** |
| S80 | None | *S. pneumoniae*, S. aureus^#^ ** | *S. pneumoniae*, S. aureus^#^, E. coli*, K. pneumoniae^#^* |

*: Pathogen exists confirmed by qRT-PCR; ^#^: Pathogen doesn’t exist confirmed by qRT-PCR
